# Ketamine Inhibits Ovarian Cancer Cell Growth by Regulating the lncRNA-PVT1/EZH2/p57 Axis

**DOI:** 10.3389/fgene.2020.597467

**Published:** 2021-03-08

**Authors:** Tao Li, Jie Yang, Ben Yang, Guoqing Zhao, Hai Lin, Qi Liu, Leiming Wang, Yingchun Wan, Hongyang Jiang

**Affiliations:** ^1^Department of Anesthesiology, China-Japan Union Hospital, Jilin University, Changchun, China; ^2^Department of Endocrinology, China-Japan Union Hospital, Jilin University, Changchun, China; ^3^Department of Ophthalmology, China-Japan Union Hospital of Jilin University, Changchun, China; ^4^Outpatient Department of Aviation University of Air Force, Changchun, China; ^5^Department of Molecular Medicine, University of Texas Health Science Center at San Antonio, San Antonio, TX, United States; ^6^Shenzhen Bay Laboratory, Gaoke International Innovation Center, The Institute of Chemical Biology, Shenzhen, China

**Keywords:** ketamine, ovarian cancer, lncRNA, p300, histone acetylation

## Abstract

Ketamine is widely used for cancer pain treatment in clinic, and has been shown to inhibit various tumor cells growth. However, the effect of ketamine on ovarian cancer cells growth and the downstream molecules has not been defined. In the present study, we found that ketamine significantly inhibited the proliferation and survival of six ovarian cancer cell lines. Moreover, ketamine induced ovarian cancer cell cycle arrest, apoptosis, and inhibited colony formation capacity. Since lncRNAs have been identified as key regulators of cancer development, we performed bioinformatics analysis of a GEO dataset and found fourteen significantly altered lncRNAs in ovarian cancer patients. We then investigated the effect of ketamine on these lncRNAs, and found that ketamine regulated the expression of lncRNA PVT1. Mechanistically, ketamine regulated P300-mediated H3K27 acetylation activation in the promoter of PVT1. Our RNA immunoprecipitation experiment indicated that PVT1 bound histone methyltransferase enhancer of zeste homolog 2 (EZH2), and regulated the expression of target gene, including p57, and consequently altered ovarian cancer cell biology. Our study revealed that ketamine could be a potential therapeutic strategy for ovarian cancer patients.

## Introduction

Ovarian cancer (OC) is the most lethal gynecologic malignancy in women (Doubeni et al., 2016; Trabert et al., 2020). There are approximately 22,240 new cases and estimated 14,070 deaths in the U.S.A. in 2018 (Reid et al., 2017; Torre et al., 2018). Because of non-specific symptoms in the early stage and the lack of effective screening methods, more than 70% of patients with ovarian cancer are in an advanced stage (FIGO stage III or IV) when diagnosed. Currently, standard treatment for ovarian cancer is surgery and chemotherapy. There are many potential new treatment options based on the modifications of standard approaches and the addition of a new biological drugs emerging from recent clinical trials (Matulonis et al., 2016). However, biological drugs and new therapeutic approaches were not shown to cure ovarian cancer, recurrence and chemotherapy resistance still cannot be ignored (Matulonis et al., 2016; Moore et al., 2018). Therefore, new therapeutic approaches are still in need.

Long non-coding RNAs (lncRNAs) are a group of RNAs that classified as ≥200 nucleotide long RNAs, and are involved in diverse molecular genetics and cellular processes, including cell proliferation, embryonic development and tumorigenesis via regulating gene expression (Wang and Chang, 2011). Recently, more and more lncRNAs are demonstrated to be dysregulated in cancer and involved in a wide range of cancer biological steps (Bartonicek et al., 2016; Evans et al., 2016; Tang et al., 2017). In ovarian cancer, studies have shown that the dysregulation of lncRNAs is frequently observed, and play a critical role in OC cell proliferation, apoptosis, cell cycle arrest, migration, invasion, and drug-resistance (Wang et al., 2019).

Ketamine, an NMDA (*N*-methyl-d-aspartate) receptor antagonist, was first approved as an anesthetic for clinical use in 1970, and is now widely used as an anesthetic, analgesic, or sedative in various clinical settings (Persson, 2013). Ketamine is often used in cancer pain treatment in patients with opiate-resistant pain because of its pronounced analgesia even in subnarcotic doses (Bredlau et al., 2013). Nevertheless, previous studies have shown that ketamine can induce dose-dependent neurotoxicity, including neuronal apoptosis and cell death in neurons and neural stem progenitor cells (Bai et al., 2013; Wang et al., 2014). Furthermore, it has been reported that ketamine regulates the proliferation and survival of several cancers, including hepatocellular carcinoma, pancreatic cancer and lung adenocarcinoma (Malsy et al., 2015; Yamaguchi et al., 2013; Zhou et al., 2018). However, the effect of ketamine on ovarian cancer cells growth and the downstream molecules remains largely unknown.

In this study, we used several pharmacologic and biochemical assays to identify the possible effect and mechanism of ketamine on OC cells. We found that ketamine inhibited OC cell growth by targeting the lncRNA-PVT1. Thus, ketamine can be considered as a possible candidate molecule for cancer therapy.

## Materials and Methods

### Cell Lines and Reagents

The human ovarian cancer cell lines OVCAR-3, SKOV3, A2780, 3AO, COC1, OV-90, and human ovarian surface epithelial cells (HOSEpiC) were purchased from Type Culture Collection of Chinese Academy of Sciences (Shanghai, China). OVCAR-3, SKOV3, A2780, and COC1 cells were maintained in RPMI1640 medium (Corning, United States) supplemented with 10% fetal bovine serum (FBS; Gibco; Thermo Fisher Scientific, Inc.), 100 U/ml penicillin and 100 μg/ml streptomycin (Thermo Fisher Scientific, Inc.) and cells were cultured at 37°C with 5% CO2. 3AO and OV-90 cells were maintained in Dulbecco’s Modified Eagle Medium (DMEM) (Corning, United States) supplemented with 10% fetal bovine serum (FBS; Gibco; Thermo Fisher Scientific, Inc.), 100 U/ml penicillin and 100 μg/ml streptomycin (Thermo Fisher Scientific, Inc.). HOSEpiC cells were maintained in ovarian epithelial cell medium (ScienCell, United States).

Ketamine was supplied by Sigma-Aldrich (United States) and dissolved in DMSO.

### Ovarian Cancer Patient Data Mining

The whole data of ovarian cancer patients were downloaded from the GEO dataset (GSE38666)^1^ (Lili et al., 2013). Data mining is implemented in the R programing language. Data were normalized by z-score in different samples. Heatmap was generated using clustering method and was used to reveal the differentially expressed ovarian cancer related lncRNAs when comparing that in normal tissues or ovarian cancer tissues.

### Cell Proliferation, Survival, and Colony Formation Assay

Cell proliferation was assessed using Sulforhodamine B (SRB) assay. Briefly, OC cells were seeded in 96-well (3,000 cells per well) and treated with indicated reagents. The cell proliferation was measured by SRB assay after 3 days treatment (Vichai and Kirtikara, 2006). Cell survival was assessed using trypan blue staining, in which dead cells were blue stained, and counted manually using hemocytometer.

For the colony formation assay, OC cells (1,500 cells/well) were seeded in 6-well plate and maintained in medium for 10–14 days. Subsequently, the colonies were fixed with 4% paraformaldehyde and stained with 0.1% crystal violet, and the number of clones was counted using an inverted microscope.

### Quantitative Real-Time PCR (QRT-PCR)

Total RNA from OC cells was isolated using RNA isolation kit (Qiagen, United States) according to the manufacturer’s protocol. iScript^TM^ Reverse Transcription Super mix kit (Bio-Rad, United States) was used for cDNA synthesis, and the samples were analyzed using SYBR Green Master Mix on a real-time PCR system (Bio-Rad). GAPDH was utilized as an endogenous calibrator control. The primer sequences used were as follows: PVT1, forward 5′-TGAGAACTGTCCTTACGTGACC-3′, Reverse 5′-AGAGCACCAAGACTGGCTCT-3′; MALAT1, forward 5′-AGAGCACCAAGACTGGCTCTGTAAC-3′, Reverse 5′-GAACAGAAGGAAGAGCCAAG-3′; LINC00092, forward 5′-CCTATGATTTGGCCTCTGGA-3′, reverse 5′-GAGAGCA GCGTTCAGGAAAC-3′; PTAR, forward 5′-ACAGATGTAAAC CAACCAGA-3′, reverse 5′-ATGCTACTGGAGACTTTAGG-3′; SnaR, forward 5′-TGGAGCCATTGTGGCTCCGGCC-3′, reverse 5′-CCCATGTGGACCAGGTTGGCCT-3′; Meg3, forward 5′-CTGCCCATCTACACCTCACG-3′, reverse 5′-CTC TCCGCCGTCTGCGCTAGGGGCT-3′; ZFAS1, forward 5′-ACG TGCAGACATCTACAACCT-3′, reverse 5′-TACTTCCAACAC CCGCAT-3′; UCA1, forward 5′-CTCTCCTATCTCCCTTCAC TGA-3′, reverse 5′-CTTTGGGTTGAGGTTCCTGT-3′; MIR4697HG, forward 5′-GAAGTGTGTGTGCAGGCTTG-3′, reverse 5′-GGAAAAGGCTCTGTCGTGGA-3′; TUG1, forward 5′-TAGCAGTTCCCCAATCCTTG-3′, reverse 5′-CACAAATTC CCATCATTCC-3′; DNM3OS, forward 5′-GGTCCTAAATTCA TTGCCAGTTC-3′, reverse 5′-ACTCAAGGGCTGTGATTT CC-3′; EWSAT1, forward 5′-GTGTCTGGCAAGGAACAC TA-3′, reverse 5′-GGTGGAGAAGAGGGACAAT-3′; HOTAIR, forward 5′-GGCAAATGTCAGAGGGTTCT-3′, reverse 5′-TT CTTAAATTGGGCTGGGTC-3′; GAS5, forward 5′-TGGTTCT GCTCCTGGTAACG-3′, reverse 5′-AGGATAACAGGTCTGC CTGC-3′; and GAPDH, forward 5′-CACCCACTCCTCCACC TTTG-3′ and reverse 5′-CCACCACCCTGTTGCTGTAG-3′. The 2-ΔΔCq method was used to calculate the relative expression levels.

### Western Blotting

Cells were lysed by radioimmunoprecipitation buffer, and 20 μg cellular protein extracts were separated in SDS-PAGE gel and was then transferred to nitrocellulose membranes (Millipore, United States). Membrane was blocked with 5% non-fat milk and incubated with antibodies against cytocrome C (1: 1,000, Abcam Biotechnology, United States), VDAC (1: 1,000, Thermo Scientific, United States), PARP1 (1: 1,000, Cell Signaling Technology, United States), or Actin (1: 5,000, Santa Cruz Biotechnology, United States) overnight at 4°. Then, the membranes were incubated with secondary antibody and the proteins were visualized using Super Signal West Pico Chemiluminescent Substrate (Thermo Scientific).

### Caspase-3/7 Activity Assay

Caspase-3/7 activity was assessed by using Apo-ONE^TM^ Homogeneous Caspase-3/7 Assay (Promega Corporation, United States) according to the manufacturer’s instruction.

### Cell Cycle Analysis

After treated with vehicle or indicated ketamine, the OC cells were harvested by trypsinization, fixed with 70% ethanol, and then retained at −20°C overnight. After washed with PBS three times, cells were resuspended in propidium iodide (PI) solution that contains RNase (100 μg/mL), and incubated in dark at room temperature for 30 min followed by a flow cytometer study.

### Transit Transfection

P300 siRNA was purchased from Sigma Aldrich (United States). Lipofectamine RNAiMAX (Invitrogen, United States) was used for transfection according to the instruction.

### Chromatin Immunoprecipitation (ChIP) Assay

SimpleChIP^®^ Enzymatic Chromatin IP Kit (Cell Signaling Technologies, United States) was used for ChIP assays according to manufacturer’s instructions; antibodies against H3K27ac (Cell Signaling Technology, United States), P300 (Cell Signaling Technology, United States) and EZH2 (Cell Signaling Technology, United States) were used for immunoprecipitation. Immunoprecipitated DNA was analyzed by QRT-PCR using the following primers: PVT1 promoter fragment 1: F: 5′-GCA GGAGAATCGCTTGAAC-3′ and R: 5′-ACAGATGTAAGAG CTGCCC-3′; fragment 2: F: 5′-GAACAAGATAACCACA TCCCAC-3′ and R: 5′-TTTCCAGAAGCCGAGTTGC-3′; fragment 3: F: 5′-TCTGGCCCTCCTATTTCAC-3′ and R: 5′-TTTCCCTGAGCCCTCTTAC-3′; fragment 4: F: 5′-CAGA GCCTACCCTCCGCT-3′ and R: 5′-CGGGGCTGGCGGGTT-3′; fragment 5: F: 5′-TCCTCCCCAATCTAAGTGCC-3′ and R: 5′-GCCAGTCACTTTCCCGTTTC-3′. P57 promoter primer: F: 5′-GGTGTCTAGGTGCTCCAGGT-3′ and R: 5′-GCACTCTCCAGGAGGACACA-3′.

### RNA Immunoprecipitation Assay

RNA immunoprecipitation (RIP) was conducted by using RNA Binding Protein Immunoprecipitation Kit (Millipore, United States) following the manufacturer’s instructions. Antibodies against EZH2 and IgG (control) (Cell Signaling Technology, United States) were used for immunoprecipitation. Immunoprecipitated RNAs were then determined by QRT-PCR analysis.

### Statistical Analysis

Data were presented as mean ± *SD* from three independent experiments. *P*-value was determined using Two-tailed Student’s *t*-test and ANOVA test. The results were illustrated with GraphPad 7. *P* < 0.05 was deemed to indicate statistical significance.

## Results

### Ketamine Inhibited OC Cells Growth

**FIGURE 1:**
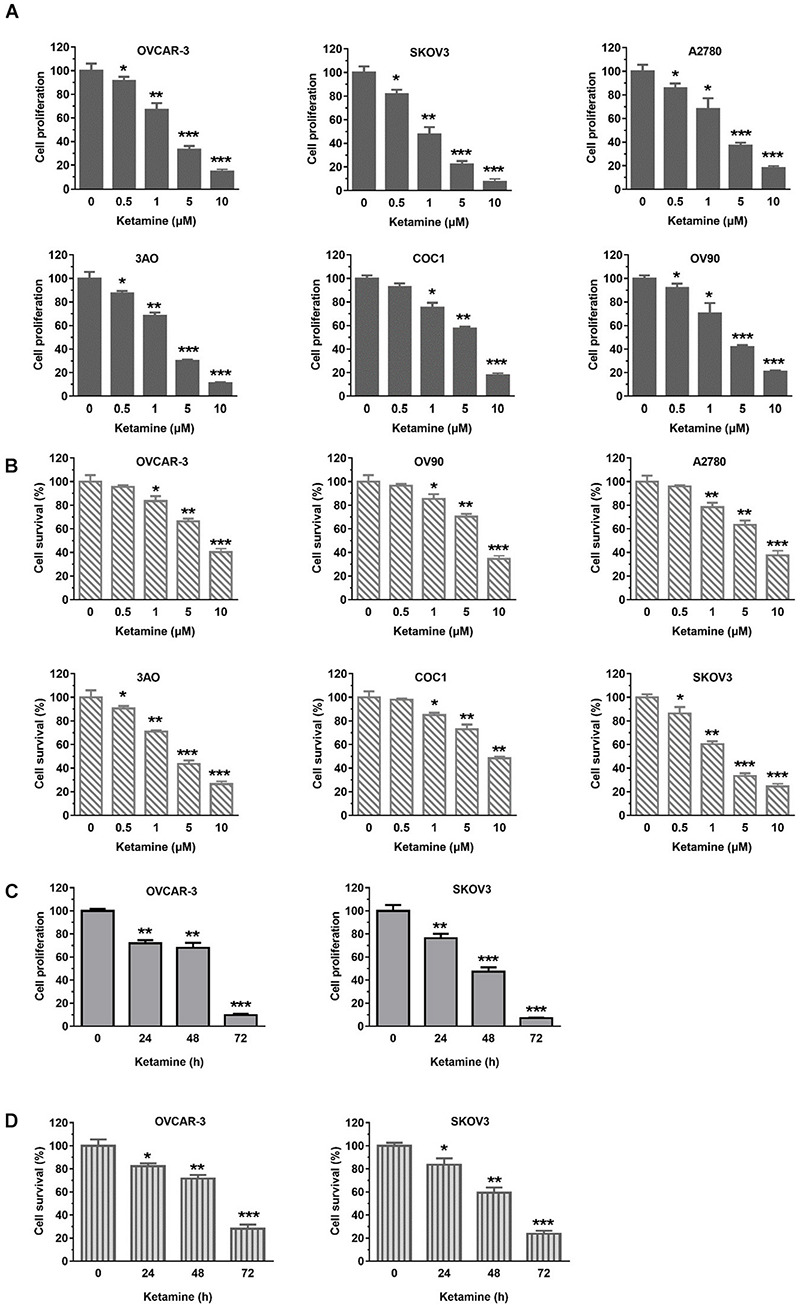
Ketamine inhibited OC cells growth. **(A,B)** Six OC cell lines were treated with indicated concentration of ketamine for 72 h, cell proliferation and survival were assessed by Sulforhodamine B (SRB) assay **(A)** and trypan blue staining **(B)** *P < 0.05, **P < 0.01, ***P < 0.001. **(C,D)** OCVAR-3 and SKOV3 cells were treated with 10 μM ketamine for indicated time. Then, cells were analyzed for proliferation **(C)** and survival **(D)**. *P < 0.05, **P < 0.01, ***P < 0.001.

o investigate the inhibitory effect of ketamine on OC cells growth, six OC cell lines (OVCAR-3, SKOV3, A2780, 3AO, COC1, OV90) were treated with indicated concentration of ketamine. As shown in Figures 1A–D, ketamine treatment significantly inhibited OC cells proliferation and survival in dose- and time-dependent manner. We next evaluated the effect of ketamine on normal human ovarian surface epithelial cells (HOSEpiC). No obviously inhibitory effect was observed after ketamine treatment (Supplementary Figure S1).

### Ketamine Regulated Cell Cycle Arrest, Cell Apoptosis, and Colony Formation Capacity in OCVAR-3 and SKOV3 Cells

**FIGURE 2:**
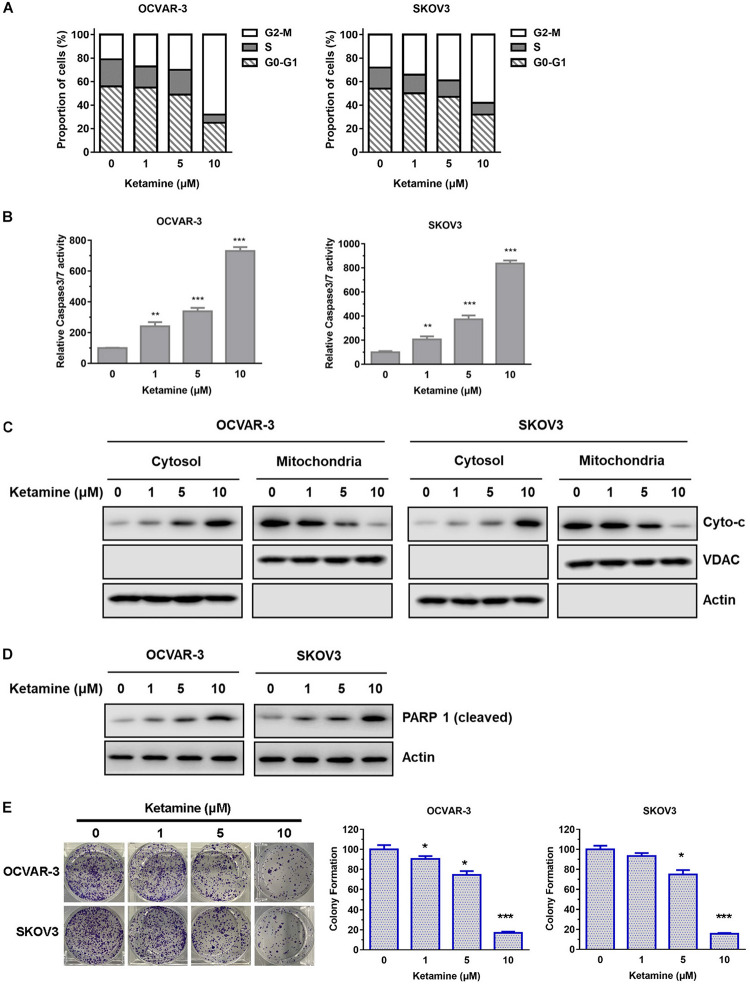
Ketamine regulated cell cycle arrest, cell apoptosis and colony formation capacity in OCVAR-3 and SKOV3 cells. **(A)** OCVAR-3 and SKOV3 cells were treated with ketamine and subsequently analyzed by PI staining to determine cell cycle phase distribution. **(B)** OCVAR-3 and SKOV3 cells were treated with ketamine, and the relative caspase-3/7 activity was measured using Apo-One homogenous caspase-3/7 assay **P < 0.01, ***P < 0.001. **(C)** The protein level of cytochrome c and VDAC in cytosol and mitochondria was analyzed by western blotting after treated with ketamine. **(D)** The protein level of cleaved PARP1 was analyzed by Western blotting after treated with ketamine. **(E)** OCVAR-3 and SKOV3 cells were performed colony formation assay after incubated with indicated concentration of ketamine. *P < 0.05, ***P < 0.001.

The inhibitory effect of ketamine was further investigated in terms of possible mechanisms and cell cycle. As shown in Figure 2A, flow cytometry results indicated that ketamine treatment caused a distinct increase in the cells arrested at G2-M phase in OCVAR-3 and SKOV3 cells. In addition, the activity of caspase3/7 was higher after ketamine treatment (Figure 2B). The ketamine treatment also elevated the disassociation of cytochrome C from mitochondria and PARP 1 cleavage in both cell lines (Figures 2C,D). Furthermore, colony formation assay results showed that the colony formation capacity of OCVAR-3 and SKOV3 cells decreased after ketamine treatment (Figure 2E). These data indicated that ketamine elevated cell cycle arrest and cell apoptosis, but decreased colony formation capacity in OCVAR-3 and SKOV3 cells.

### LncRNAs Were Dysregulated in Ovarian Cancer Patients

**FIGURE 3:**
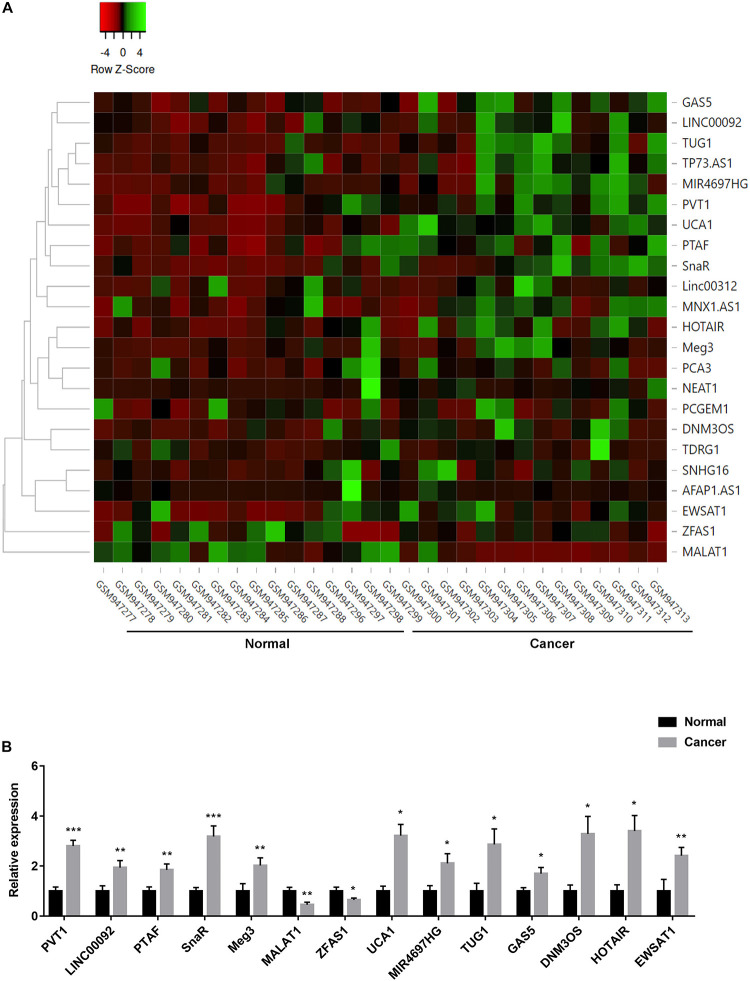
Expression of lncRNAs in ovarian cancer patients. **(A)** Heatmap represented the differential expression of lncRNAs in tumor and normal tissues in ovarian cancer patients. Data were normalized by *z*-score in different samples. **(B)** The expression of fourteen lncRNAs in tumor and normal tissues *P < 0.05, **P < 0.01, ***P < 0.001.

ncRNAs have been reported to play important role in controlling ovarian cancer cell proliferation and survival (Wang et al., 2019). To investigate the differential lncRNA expression in ovarian cancer patients, we did bioinformatics analysis of a GEO dataset (GSE38666). The expression level of lncRNAs that have been reported to relate to ovarian cancer was compared between ovarian cancer and normal tissues, and demonstrated using heatmap and volcano plot as shown in Figure 3A and Supplementary Figure S2. Fourteen lncRNAs (PVT1, LINC00092, PTAF, SnaR, Meg3, MALAT1, ZFAS1, UCA1, MIR4697HG, TUG1, GAS5, DNM3OS, HOTAIR, and EWSAT1) were found significantly dysregulated, including several lncRNAs that has been shown to play important role in other tumors, such as PVT1, MALAT1, TUG1, GAS5, and HOTAIR (Figure 3B; Wang et al., 2019).

### Ketamine Regulated lncRNA PVT1 Expression via P300 in OCVAR-3 and SKOV3 Cells

**FIGURE 4:**
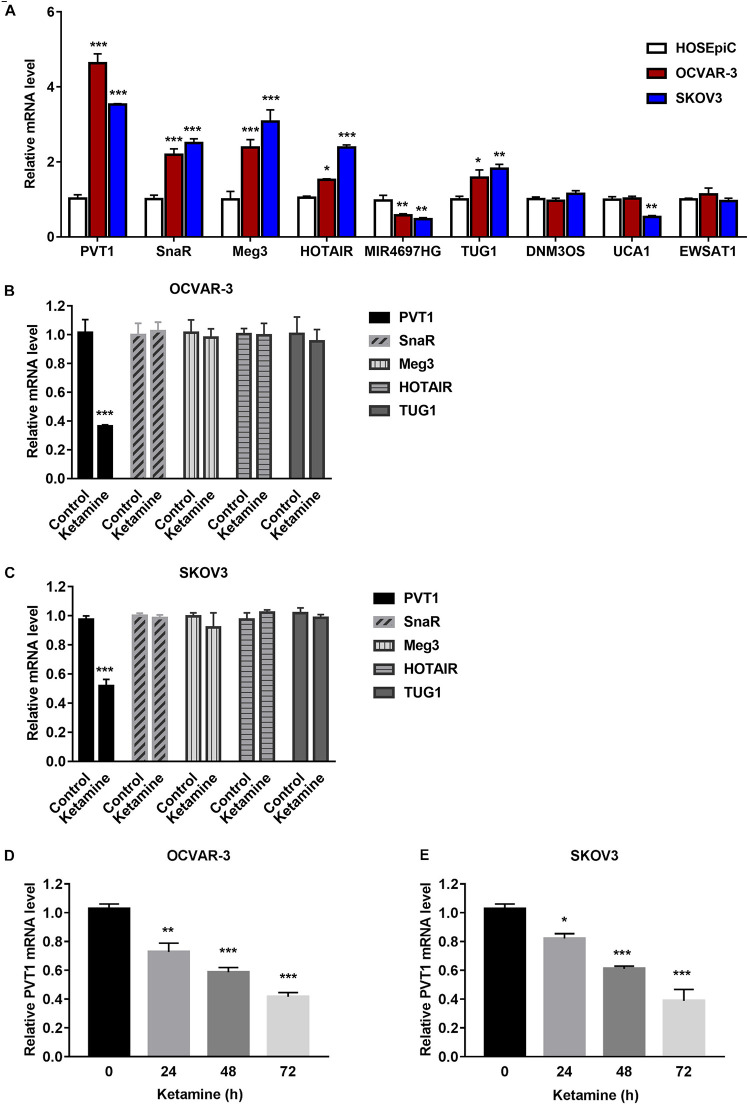
Ketamine regulated lncRNA PVT1 expression in OCVAR-3 and SKOV3 cells. **(A)** The expression level of lncRNAs PVT1, SnaR, Meg3, HOTAIR, MIR4697HG, TUG1, DNM3OS, UCA1, and EWSAT1 were analyzed by QRT-PCR in OCVAR-3 and SKOV3 and parental cells *P < 0.05, **P < 0.01, ***P < 0.001. **(B,C)** The expression level of lncRNAs PVT1, SnaR, Meg3, HOTAIR, and TUG1 were analyzed by QRT-PCR after treated with ketamine in OCVAR-3 **(B)** and SKOV3 cells **(C)** ***P < 0.001. **(D,E)** OCVAR-3 **(D)** and SKOV3 **(E)** cells were treated with 10 μM ketamine for indicated time, the expression level of lncRNAs PVT1 was analyzed by QRT-PCR *P < 0.05, **P < 0.01, ***P < 0.001.

To further confirm the results from bioinformatics analysis, we evaluated the expression level of nine lncRNAs (PVT1, SnaR, Meg3, HOTAIR, MIR4697HG, TUG1, DNM3OS, UCA1, and EWSAT1), which showed more obvious differences in expression based on Figure 3B, in ovarian cancer cell lines. Compared with parental lines, lncRNAs PVT1, SnaR, Meg3, HOTAIR, and TUG1 were significantly overexpressed in OCVAR-3 and SKOV3 cells (Figure 4A), which is consistent with previous report (Wang et al., 2019). Interestingly, ketamine significantly decreased the expression level of lncRNA PVT1, but had no effect on other lncRNAs in OCVAR-3 and SKOV3 cells (Figures 4B,C). Results in Figures 4D,E further confirmed that ketamine regulated the expression of lncRNA PVT1 in a time-dependent manner.

**FIGURE 5:**
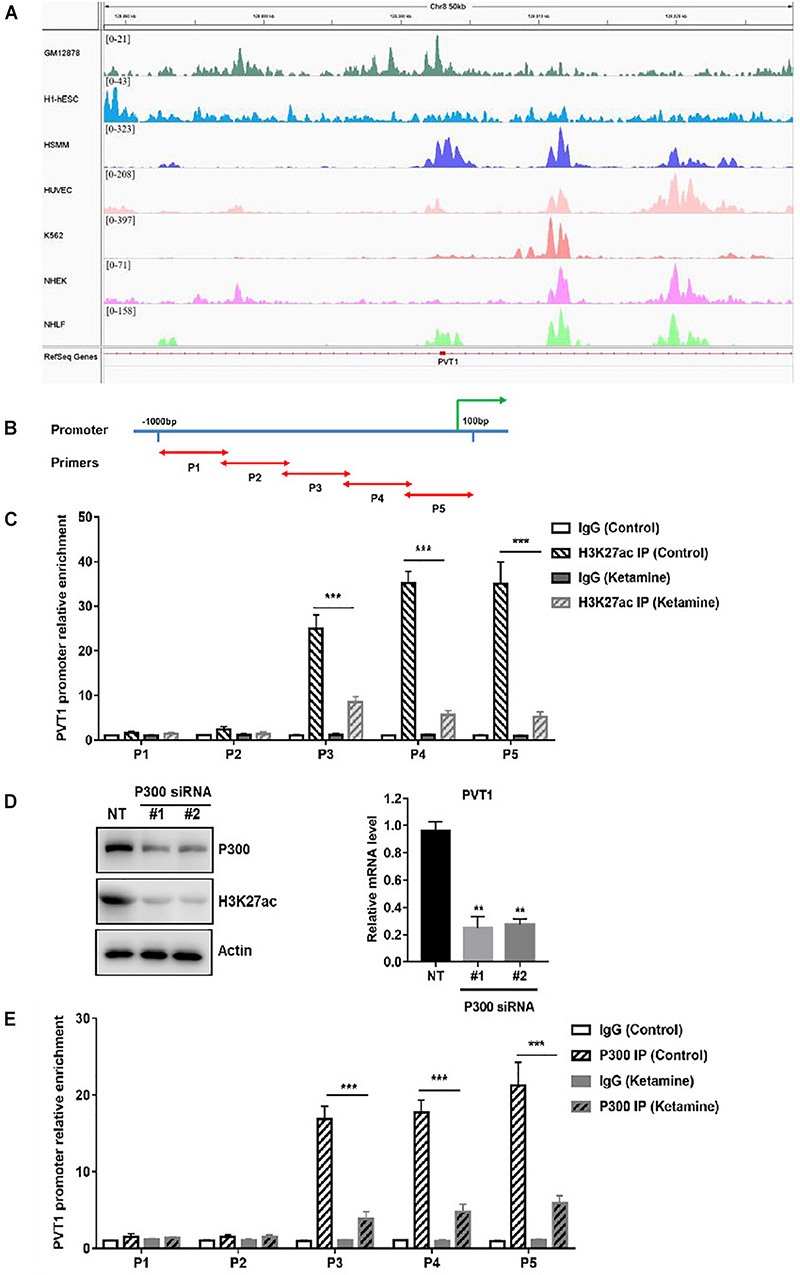
Ketamine regulated P300 mediated PVT1 transcription. **(A)** Visualization of H3K27ac enrichment of 7 cell lines around TSS of PVT1. **(B)** Primers were designed to cover 5 regions within∼1 kb promoter region of PVT1. **(C)** OCVAR-3 cells were treated with 10 μM ketamine for 48 h and ChIP assay was performed to detect enrichment of H3K27 acetyl marks on PVT1 promoter ***P < 0.001. **(D)** OCVAR-3 cells were incubated with P300 siRNA for 72 h, protein level of P300 and H3K27ac, and PVT1 level were analyzed by western blotting and QRT-PCR, **P < 0.01. **(E)** OCVAR-3 cells were treated with 10 μM ketamine for 48 h and ChIP assay was performed to detect the recruitment of P300 on PVT1 promoter.

To explore the mechanism of PVT1 overexpression in OC, we first analyzed the modification in the promoter of PVT1. Abundant H3K27 acetylation (H3K27ac) signals were found in the promoter region of PVT1, which suggested that PVT1 might be regulated by histone acetylation (Figure 5A). To confirm that, we performed chromatin-immunoprecipitation assay (ChIP) using H3K27ac antibody and primers covering 5 regions within∼1kb promoter region (Figures 5B,C). The results demonstrated that H3K27ac marks are highly enriched at the PVT1 promoter regions 3–5, and this enrichment was significantly decreased after treatment with ketamine (Figure 5C). H3K27ac is known to be catalyzed by the P300/CBP complex (Raisner et al., 2018). We then treated the OCVAR-3 with P300 specific siRNA, and the results showed that H3K27ac marks and PVT1 expression was significantly decreased (Figure 5D). Consistent with these findings, P300 was recruited to PVT1 promoter regions 3–5, and this recruitment was significantly decreased after ketamine treatment (Figure 5E). These data indicated that ketamine regulated lncRNA PVT1 expression via P300 mediated histone acetylation.

### Ketamine Regulated p57 Expression via EZH2 in OCVAR-3 and SKOV3 Cells

**FIGURE 6:**
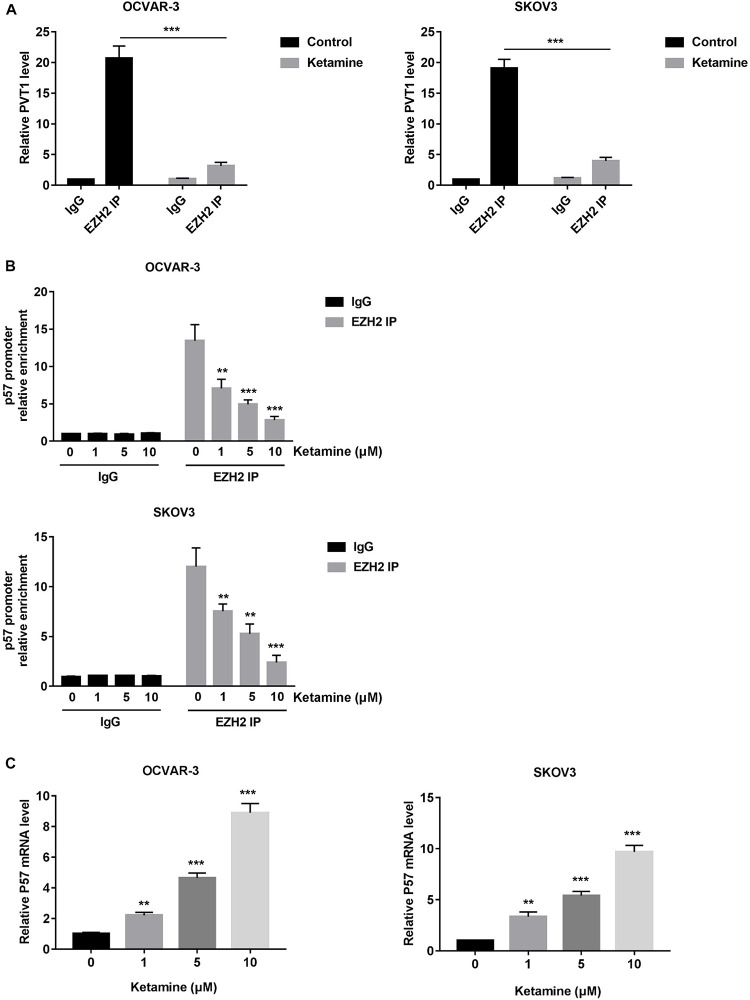
Ketamine regulated p57 expression via EZH2 in OCVAR-3 and SKOV3 cells. **(A)** OCVAR-3 and SKOV3 cells were treated with 10 μM ketamine for 48 h and the interaction between PVT1 and EZH2 was analyzed by RNA immunoprecipitation assay. **(B)** OCVAR-3 and SKOV3 cells were treated indicated concentration of ketamine for 48 h, the recruitment of EZH2 on p57 promoter was analyzed by ChIP assay **P < 0.01, ***P < 0.001. **(C)** OCVAR-3 and SKOV3 cells were treated indicated concentration of ketamine for 72 h, the mRNA level of p57 was analyzed by QRT-PCR **P < 0.01, ***P < 0.001.

Enhancer of zeste homolog 2 (EZH2), a subunit of the polycomb repressive complex 2, was reported to contribute to the deregulation of OC cell growth. In addition, PVT1 was shown to bind EZH2 and improve its stability in hepatocellular carcinoma (Guo et al., 2018). We next examine the association of PVT1 and EZH2 in OC cell by performing RIP assay. As shown in Figure 6A, PVT1 bound EZH2 in OCVAR-3 and SKOV3 cells, and this interaction was significantly decreased after ketamine treatment. More importantly, the recruitment of EZH2 to the target gene-p57 promoter region was significantly inhibited by ketamine, and consequently, the expression level of p57 was significantly increased in OCVAR-3 and SKOV3 cells (Figures 6B,C).

## Discussion

NMDA receptors are chiefly found within the central nervous system in normal tissues and are involved in synaptic plasticity and memory function. However, NMDA receptors are often found expressed in cancer cells, including glioma, oral squamous cell carcinoma, prostate cancer, osteosarcoma and gastric cancer (Aronica et al., 2001; Choi et al., 2004; Abdul and Hoosein, 2005; Kalariti et al., 2005; Liu et al., 2007). Given administration of glutamate antagonists inhibits the growth of cancer cells derived from brain, thyroid, colon, breast and lung tumors, NMDA receptors are considered to play important role in cancer cell growth (Rzeski et al., 2001; Stepulak et al., 2005). Ketamine is one of the most common NMDA receptor antagonists and often used for cancer pain treatment in clinic. Inhibitory effect of ketamine on cell growth has been reported in various cancers, including hepatocellular carcinoma, pancreatic cancer, lung adenocarcinoma and colorectal cancer (Yamaguchi et al., 2013; Malsy et al., 2015; Zhou et al., 2018; Duan et al., 2019). Although knowledge of the detailed mechanisms is limited, FOXO/TXNIP pathway, CD69 and VEGF were believed involved. As NMDA receptors are found expressed in human ovarian cancer tissues and human ovarian cancer cell lines (North et al., 2015), we assumed that ketamine might regulate the growth of ovarian cancer cells. In this study, we found that ketamine had significant anti-proliferative effect against ovarian cancer cells (Figure 1). The inhibitory effect caused by ketamine may result from induction of apoptosis and arrest of cell cycle at G2-M (Figure 2).

To understand the mechanisms of action of ketamine, we analyzed the expression level of long non-coding RNAs (lncRNAs). LncRNAs are considered as new and valuable molecules that are involved in tumorigenesis. Several lncRNAs have been reported to regulate OC cell growth, including PVT1, MALAT1, TUG1, HOTAIR, and GAS5 (Ozes et al., 2016; Hosseini et al., 2017; Martini et al., 2017; Long et al., 2019; Gu et al., 2020). In order to find out which lncRNA might be ketamin related, we performed bioinformatics analysis of a GEO dataset obtained from OC patients. Fourteen lncRNAs were dysregulated in OC patients, and five of them were significantly increased in OC cell lines (Figure 3). We then evaluated the expression of these lncRNAs after ketamine treatment. Among these lncRNAs, only lncRNA PVT1 was significantly decreased after ketamine treatment in OC cells (Figure 4). Although lncRNA PVT1 was reported up-regulated in OC cells in several studies, none of them investigated the mechanism of dysregulation. Here, we analyzed the modification, specifically histone acetylation, in the PVT1 promoter by using UCSC genome bioinformatics site. Abundant H3K27ac signals are found near the transcription starting site (TSS) in the promoter of PVT1. Our ChIP assay confirmed that H3K27ac marks are highly enriched at the PVT1 promoter regions 3-5, which is closer to TSS. Interestingly, the treatment of ketamine significantly decreased the enrichment of H3K27ac marks in the promoter of PVT1. Since H3K27ac is widely known to be catalyzed by the P300/CBP complex, we then wonder whether ketamine could regulate P300 function. Our results indicated that the recruitment of P300 to the PVT1 promoter was significantly inhibited by ketamine treatment (Figure 5).

In order to further investigate the functional role of ketamine in OC, we sought to find out the binding partner of PVT1. EZH2, a member of polycomb repressive complex 2 (PRC2), is commonly involved in transcriptional repression in cancer cells. In ovarian cancer, EZH2 upregulation has been widely established. The overexpression of EZH2 promotes cell proliferation and invasion, inhibits apoptosis and enhances angiogenesis in epithelial ovarian cancers (Li et al., 2010;To understand Lu et al., 2010). PVT1 was reported to bind EZH2 and improve the EZH2 protein stability in hepatocellular carcinoma (Guo et al., 2018). In consistent with this report, our RIP results confirmed the interaction between PVT1 and EZH2, and this interaction was significantly inhibited by ketamine treatment. One important mechanism by which EZH2 promotes OC cell growth is by regulating p57, a cyclin dependent kinase inhibitor that regulates tumor cell transcription, differentiation, apoptosis, and migration (Guo et al., 2010, 2011). Not surprisingly, ketamine treatment decreased the recruitment of EZH2 to the promoter of p57, and the expression level of p57 was significantly increased (Figure 6).

In summary, all of our results suggest that ketamine significantly inhibited the proliferation and survival of ovarian cancer cells. Mechanistically, ketamine inhibited lncRNA PVT1 expression, the recruitment of EZH2 to p57 promoter, and subsequently increased the tumor suppressor gene-p57 expression. These results suggest a rational and novel treatment strategy for ovarian cancer patients.

## Accession Codes

Raw and processed lncRNA datasets are publicly available in GEO under accession number GSE 38666.

## Data Availability Statement

The original contributions presented in the study are included in the article/Supplementary Material, further inquiries can be directed to the corresponding author/s.

## Author Contributions

TL, JY, BY, GZ, HL, QL, and LW carried out experiments and analyzed the data. LW verified the analytical methods and supervised the findings of this work (for the Figure 5). YW and HJ guided experiment. All authors discussed the results and contributed to the final manuscript.

## Conflict of Interest

The authors declare that the research was conducted in the absence of any commercial or financial relationships that could be construed as a potential conflict of interest.

## Footnotes

1 https://www.ncbi.nlm.nih.gov/geo/query/acc.cgi?acc=GSE38666
