# Supplementary material for: Ketamine Inhibits Ovarian Cancer Cell Growth by Regulating the lncRNA-PVT1/EZH2/p57 Axis
Source: Front Genet. 2021 Mar 8;11:597467. doi: 10.3389/fgene.2020.597467 (PMC7982774; doi:10.3389/fgene.2020.597467)
Supplement: Supplementary file 1 [file Presentation_1.pdf]

Supplementary figure

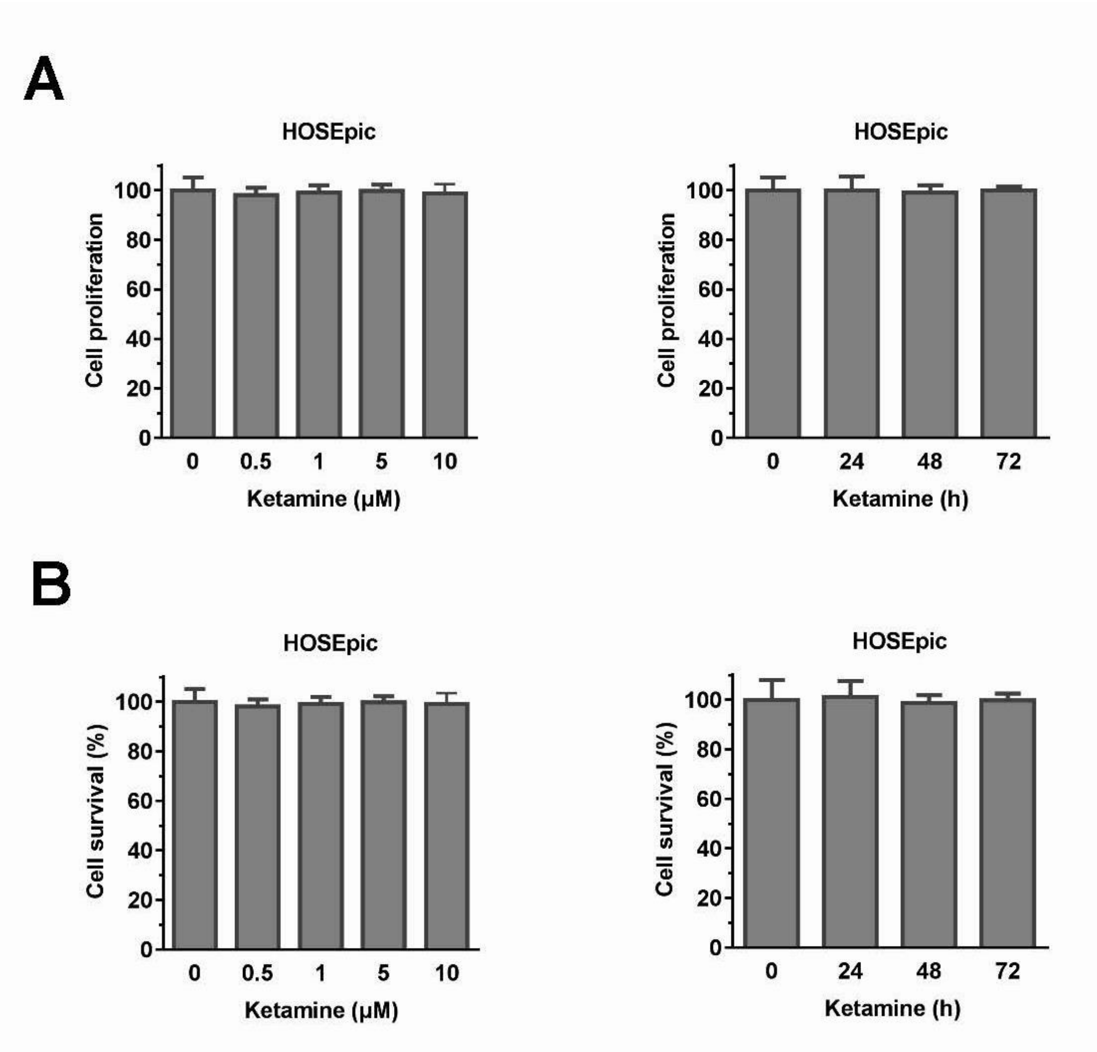

Supplemental figure legends

Figure S1. Effect of ketamine on normal human ovarian surface epithelial cells.

(A, B) Human ovarian surface epithelial cells (HOSEpic) were treated with indicated concentration of ketamine for indicated time, cell proliferation and survival were assessed by Sulforhodamine B (SRB) assay (A) and trypan blue staining (B).

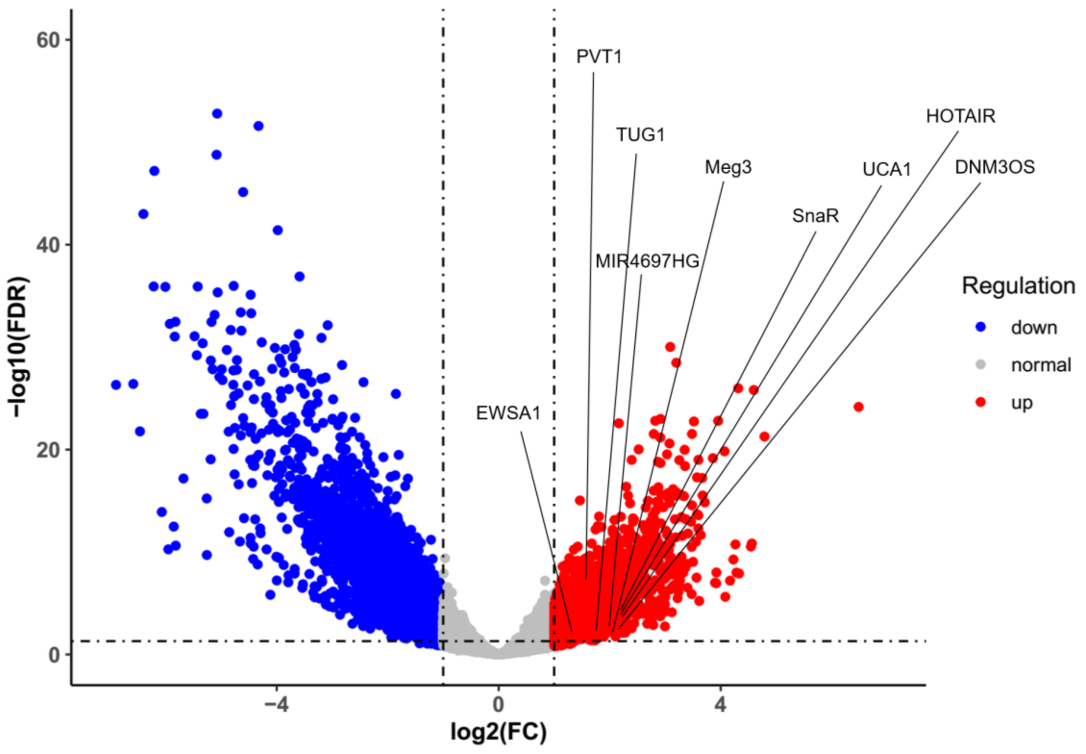

Figure S2. Differentially expressed genes in ovarian cancer patients.

Volcano plot showing up-regulated and down-regulated genes identified from RNA-Seq experiments using human. A change is considered significantly if the change is >2-fold with a p-value <0.05. IncRNA, related to ovarian cancer, with significant change were indicated.
